# Diagnosing Norms Surrounding Sexual Harassment at a Jordanian University

**DOI:** 10.3389/fsoc.2021.667220

**Published:** 2021-07-26

**Authors:** Irina Bergenfeld, Beniamino Cislaghi, Kathryn M. Yount, Aida A. Essaid, Jude Sajdi, Rand Abu Taleb, Grace L. Morrow, Janice S. D’Souza, Rachael A. Spencer, Cari Jo Clark

**Affiliations:** ^1^ Rollins School of Public Health, Emory University, Atlanta, GA, United States; ^2^ London School of Hygiene and Tropical Medicine, London, United Kingdom; ^3^ Information and Research Center, King Hussein Foundation, Amman, Jordan; ^4^ Department of Behavioral, Social, and Health Education Sciences, Rollins School of Public Health, Emory University, Atlanta, GA, United States

**Keywords:** Jordan, sexual harassment, university, social norms, gender norms

## Abstract

Sexual harassment (SH) is a form of gender-based violence (GBV) that negatively impacts women’s physical, mental, social, and financial well-being. Although SH is a global phenomenon, it also is a contextualized one, with local and institutional norms influencing the ways in which harassment behavior manifests. As more women attend institutions of higher education in Jordan, these women are at increased risk of experiencing SH in university settings, with potential implications for their health and future employment. Social norms theory, which examines the informal rules governing individual behavior within groups, has been a useful framework for understanding and developing interventions against GBV globally. We sought to apply a social-norms lens to the understanding and prevention of SH at a Jordanian university. To gain a comprehensive and nuanced picture of social norms surrounding SH, we collected qualitative data using three complementary methods: focus group discussions (n = 6) with male and female students (n = 33); key informant interviews with staff and faculty (n = 5); and a public, participatory event to elicit anonymous short responses from students (n = 317). Using this data, we created a codebook incorporating social-norms components and emergent themes. As perceived by participants, SH was unacceptable yet common, characterized as a weak norm primarily because negative sanctioning of harassers was unlikely. Distal norms related to gender and tribal affiliation served to weaken further norms against SH by blaming the victim, preventing reporting, discouraging bystander intervention, and/or protecting the perpetrator. The complexity of the normative environment surrounding SH perpetration will necessitate the use of targeted, parallel approaches to change harmful norms. Strengthening weak norms against SH will require increasing the likelihood of sanctions, by revising university policies and procedures to increase accountability, increasing the acceptability of bystander intervention and reporting, and fostering tribal investment in sanctioning members who harass women. Creating dialogue that emphasizes the harmful nature of SH behaviors and safe spaces to practice positive masculinity also may be an effective strategy to change how male students interact in the presence of peers. Any social norms change intervention will need to consider the various reference groups that dictate and enforce norms surrounding SH.

## Introduction

Sexual harassment (SH), or unwelcome conduct of a sexual or gendered nature (National Academies of Sciences, E., & Medicine, 2018; Smith, 1980), is a global issue with negative impacts on women’s physical, mental, social, and financial well-being (Bondestam and Lundqvist, 2020). Individuals who experience SH are more likely to experience negative psychosocial outcomes including anxiety, depression, and post-traumatic stress disorder (Fitzgerald et al., 1997; Huerta et al., 2006; Muazzam et al., 2016; Willness et al., 2007). Experiences of SH in university settings carry additional consequences for women, including decreased academic performance and even withdrawal from the academic environment (Pinchevsky et al., 2019), with potentially long-term implications for future employment prospects (McLaughlin et al., 2017).

Although SH is a global phenomenon, it also is a highly contextualized one, with local and institutional norms influencing the ways in which harassment behavior manifests (Tyler and Boxer, 1996).

SH has been studied through the lens of various disciplines, including psychology, sociology, law, ethics, and feminist theory (Pina et al., 2009). This disciplinary diversity has produced several competing models to explain SH perpetration, including biological, sex role spillover, sociocultural, organizational, and multifactorial theories incorporating elements of one or more other models (Pina et al., 2009; Whaley and Tucker, 1998). However, scholars generally agree that broader social factors, including workplace culture or societal expectations around gender, play a key role in determining an individual’s likelihood of perpetrating SH in a given situation. Pryor and colleagues’ 1993 work describing the characteristics of men deemed “likely to harass” noted that these men were more likely to perpetrate SH where it was perceived as a normative behavior in the workplace and where sanctions were unlikely, especially when perceived normative signals arose from office leadership (Pryor et al., 1993). Similar trends have been observed among adolescents, who are more likely to harass when those in their school-based social networks also harass (Jewell et al., 2015). The perception that peers and/or supervisors are unwilling to impose negative sanctions for harassment has been shown to increase SH and to reduce reporting across a variety of workplace (Murdoch et al., 2009; Hardies, 2019) and academic (National Academies of Sciences, E., & Medicine, 2018) contexts, further decreasing the likelihood that perpetrators will receive negative sanctions. In many of these studies, supervisors emerged as key reference groups for norms surrounding SH (Murdoch et al., 2009; Tenbrunsel et al., 2019).

On a societal level, gender norms that reinforce inequality between men and women have long been implicated in SH and other forms of sexual violence. Qualitative research among men and boys from various contexts has uncovered strikingly similar themes, including naturalization of men’s sexual desire, perpetration of SH to prove one’s masculinity, minimization of the harm done to women, and victim blaming (Fan et al., 2018; Heilman and Barker, 2018; Henry, 2017; Nahar et al., 2013; Zietz and Das, 2018). In societies with a higher degree of homosociality and sex segregation, normative pressure on women to avoid male spaces also may exacerbate women’s fears of victim blaming (Henry, 2017; Nahar et al., 2013; Zietz and Das, 2018). Scholars in Europe (Galdi et al., 2014; Zambelli et al., 2018) and the Arab world (Chafai, 2017) have documented the ways in which media reinforce the objectification and inferior status of women, and how these contribute to men’s perpetration of SH. Such broad similarities in findings across the range of disciplines that study SH suggest that social norms theory is an ideal framework to examine the ways in which gender and social norms contribute to this phenomenon, yet few studies to date have applied this lens to interrogate SH.

Social norms are “the unwritten rules governing acceptable behavior in a group” enforced by “relevant others” within that group (Mackie et al., 2015). Gender norms, which have been a focus of health and development research, are a subset of social norms “defining acceptable and appropriate actions for women and men in a given group or society” (Cislaghi and Heise, 2020). In recent years, social-norms change interventions have gained traction in global health as a means to change entrenched harmful practices into healthy ones. However, the pathways through which norms influence individual behavior rarely are straightforward, being complicated by the influence of individual attitudes and beliefs, as well as by situational and environmental factors (Cislaghi and Heise, 2018b). Cislaghi and Heise (2018a) have hypothesized four main avenues of normative influence on behavior: the degree to which the norm is interdependent, the degree to which compliance is detectable, the strength and likelihood of sanctions, and the proximity of the norm to the behavior. Each of these factors influences the relative strength of the norm and has implications for the design of interventions. For example, intervening to change an interdependent norm requires group coordination to achieve the collective goal that the previous harmful norm had achieved, while in the case of weaker norms, strengthening sanctions or giving individualized normative feedback may be an appropriate strategy (Cislaghi and Heise, 2018a).

Social norms theory has been used to understand and to design interventions to address gender-based violence (GBV). Efforts to reduce the practice of female genital cutting using social norms are a notable focus of this approach (Hayford et al., 2020; Insight, 2010), inspiring practitioners to apply social norms change to combat other forms of GBV, including child marriage (Karim et al., 2016; Kathryn M Yount et al., under review), intimate partner violence (Clark et al., 2018; Kyegombe et al., 2014), and campus sexual assault (Fabiano et al., 2003; Kathryn M Yount et al., 2020). Challenges and successes of social-norms change interventions have highlighted certain best practices in using such an approach to combat GBV, including 1) using formative research to tailor programming to the local context, 2) identifying and enabling positive behaviors to replace harmful ones, and 3) engaging community leaders and other influential reference groups (Paluck et al., 2010; WHO, 2009). Recently, there also has been greater acknowledgement that engagement of men and boys as allies (and not merely as perpetrators of violence) in social norms change is critical to the success of such interventions (Fabiano et al., 2003; Jewkes et al., 2015).

While social norms theory has been applied to GBV prevention research more broadly, the use of social-norms change interventions to reduce perpetration of SH, particularly outside of Europe and the United States, has received less attention. One study from the Netherlands evaluating a school-based intervention to reduce SH found that perceived social norms related to students’ ability to reject SH was sustained at six-month follow-up, while other main effects of the intervention were not (de Lijster et al., 2016).

Our study aimed to gain a nuanced understanding of the normative landscape surrounding SH at a university in Jordan and to use these findings to inform targeted social-norms change interventions appropriate to the Jordanian university context. Using Cislaghi and Heise’s avenues of normative influence as a framework by which to characterize norms to and to optimize norms change, we characterize intersecting proximal and distal norms influencing the perpetration and reporting of SH on the university campus.

## Methods

This analysis is based on formative data from a larger mixed-methods study aimed at understanding the scope, definition, and causes of SH on campus from the perspective of students and university employees. The parent study was approved by Emory University’s Institutional Review Board (IRB00099940), which covered the study site under a reliance agreement. We used three complementary qualitative approaches: single-sex focus group discussion (FGDs) with students; a participatory, public data collection event known as a FADFED (Jordanian slang for “let it out”) (What is FADFED?, 2013), and key informant interviews (KIIs) with university employees. Each of these data collection methods uncovered different aspects of the SH construct and/or perspectives on SH, allowing for validation and triangulation of results. The FADFED, while not explicitly norms focused, was intended to elicit student buy-in for the research; to sample a broad range of student perspectives on the definition, scope, and causes of SH; and to inform item development for future data collection. FGD guides were developed within a Social Norms Analysis Plot framework, enabling the research team to more comprehensively interrogate students’ normative and empirical expectations surrounding SH, the key reference groups guiding SH behaviors, any possible sanctions resulting from norms violation, and any exceptions to common norms. Finally, KIIs sought to gain an institutional perspective from administrator and staff, including the scope and causes of SH, institutional response, and normative barriers to addressing the problem. Table 1 summarizes each strategy, applicable frameworks as appropriate, participant eligibility, objectives, advantage, limitations, and timeframes of administration.

**TABLE 1:** Phases of qualitative data collection.

Approach	Theoretical Framework	Objectives	Timeframe	Sample	Advantages and limitations
Key informant interviews	Culture of respect evaluation National Association of Student Personnel Administrators (2017)	Define scope of SH, assess existence existing policies and available sources of assistance, and assess factors that will influence implementation (e.g., preparedness of administration officials to address SH), and opportunities to influence university policy and support services	August 2018	Five male staff and faculty administrators whose insights or position make them an essential source of information or support	Advantages: Highlights employee/institutional perspective. Purposive sampling highlights a range of institutional roles.
					Limitations: Institutional actors may feel pressured to give favorable views of their employers. Small sample size
FADFED	None	To understand range of perceptions on the issue of SH in order to begin to define its scope, perceived causes and consequences, language used to describe it, and to create a public signal that the issue is being assessed on campus	September 2018	75 male students and 242 female students	Advantages: Large sample and sampling method provides a diversity of responses. Participatory aspect encourages student buy-in to the research. Anonymity reduces social desirability bias
					Limitations: Brief responses do not allow for probing or follow-up
FGDs	Social norms theory and diagnosis Cislaghi and Heise (2016)	Determine the content, scope and boundaries of the construct and diagnosis relevant norms and reference groups associated with it	August––September 2018	21 male students in 3 FGDs and 12 female students in 3 FGDs	Advantages: Group setting with variety of activities elicits thick, rich data on various aspects of social norms
					Limitations: Small, non-random sample limits generalizability. Social desirability bias is a risk

### Eligibility and Recruitment

Our study population included currently enrolled undergraduate- and graduate-level university students, as well as administrators and staff members who had been employed at the university for at least 3 years. For recruitment, we followed the Ethical and Safety Recommendations for Intervention Research on Violence Against Women developed by the World Health Organization (Hartmann and Krishnan, 2016). These guidelines included giving FGD participants the opportunity to provide consent at multiple points in time and providing referrals to support services for violence survivors. All recruitment materials have been included in Supplementary Material S1.

We recruited initial student participants for two FGDs in August 2018 using student Facebook groups and posters on campus. Recruitment materials briefly described the project aims, time commitment, compensation, and key staff to contact for more information. Due to recruitment challenges, snowball sampling (Biernacki and Waldorf, 1981) was used to recruit additional participants for four subsequent FGDs, conducted in September 2018. Faculty and staff considered to be key stakeholders in that they were directly involved in university response to SH were identified by a knowledgeable senior study team member and invited to participate *via* email. Students who participated in the FADFED were recruited on the spot by research team members as they passed through the event space. All students who passed through this campus event space during a six-hour window were approached and offered a one-page description of the project, including research aims and key staff contact information.

### Data Collection

Following best practices when conducting GBV research, FGDs (n = 6) were conducted in sex-separate groups in the Arabic language by researchers of the same gender. Each FGD lasted approximately 90 min. KIIs were conducted in Arabic at a private location of the interviewee’s choosing and lasted approximately 60 min. Both FGDs and KIIs were recorded, transcribed verbatim, and translated into English. The FADFED, which took place at a high-traffic campus location over a period of about 6 h, was conducted by giving participants a brief introductory prompt and allowing them to submit written responses on index cards, which were then collected in a clear box. Participant gender was identified by the use of colored pens. FADFED responses were collated on a spreadsheet in the original Arabic and later translated into English. All researchers were trained in qualitative methods and research ethics, including considerations specifically related to GBV research.

### Measures

The FADFED was designed to gather short, open-ended responses to begin to define the scope, perceived causes and consequences, and language used to describe SH. The inquiry intentionally was kept very broad to garner the widest range of insights possible on the construct, and was therefore not based on any guiding theoretical framework. Participants were asked to provide brief responses to two questions: 1) “What do you understand when you hear the word “harassment”” and 2) “In your opinion, what are the forms of harassment that you think exist at the university campus?”. Student responses to the FADFED were used to guide item development in subsequent data collection exercises.

Semi-structured FGDs examined the content, scope, and boundaries of the SH construct and diagnosed relevant norms and reference groups associated with SH perpetration and reporting. The FGD guide consisted of three phases of inquiry: 1) a free listing exercise to identify the scope of perceived forms of SH, 2) a social mapping exercise to develop the content, boundaries and social norms associated with the practice, and 3) a series of vignettes to examine social norms in context. The social mapping exercise asked participants to map visually SH occurrences on campus as a group and to discuss questions such as, “Who are the victims/perpetrators?,” “Where does (SH) happen on campus?,” “Who is likely to witness (SH)?,” and “Whose opinion on the acceptability of the behavior matters to the perpetrator?”. The vignettes presented participants with hypothetical scenarios in which a woman is harassed on campus and asked participants to discuss questions designed to diagnose normative and empirical expectations, sanctions, and reference groups.

KII guides were developed based on the Culture of Respect framework recommendations for measuring and reducing sexual violence on college campuses (National Association of Student Personnel Administrators, 2017). Interviews assessed the scope of SH on campus, perceived causes of SH, existence of policies and available sources of assistance available to faculty/staff and students who are harassed, factors related to implementation of these policies, and opportunities to improve university response and support services.

Examples of questions from the KIIs included, “How difficult is it to file a complaint for sexual harassment?” and “What sanctions are levied if a person is found guilty of harassing another on campus?”. Although KII guides were not designed specifically to elicit information on social norms, descriptions of the normative climate emerged in the course of the interviews. All qualitative tools used in this study are available in Supplementary Material S2.

### Analysis

Our analytic method was guided by principles of thematic analysis (Clarke, 2003) incorporating elements of a modified grounded theory approach (Hennink et al., 2020). Firstly, five team members, including the principal investigator, drafted a codebook based off a thorough reading of four FGDs. This draft codebook featured one deductive code for types of SH based on Fitzgerald’s tripartite model (Fitzgerald et al., 1995), but was otherwise formulated using inductive codes derived entirely from the data. Codes emerging from student narratives included Tribalism, Wasta,^1^ and Institutional Factors. The draft codebook was iteratively revised through weekly discussions involving the analytic team, incorporating feedback from the broader research team in Jordan. As FGD, FADFED, and KII data became available, emergent themes from these data sources were incorporated into the existing coding framework *via* constant comparison (Glaser and Strauss, 2017; Hennink et al., 2020), and all data sources were coded using this same coding framework. Finally, for the social norms analysis, we applied systematic coding (Glaser and Strauss, 2017) using codes based on the Social Norms Analysis Plot framework (Descriptive Norms/Empirical Expectations, Injunctive Norms/Normative Expectations, Reference Groups, Exceptions, and Sanctions) (Cislaghi and Heise, 2016; Costenbader et al., 2019).

Two teams of two coders coded transcripts using MAXQDA 18 (VERBI Software, 2019). An intercoder reliability (ICR) test on a 10% subsample of each data source was performed to ensure a kappa of >0.7 before coding the full data set (McHugh, 2012). Post-ICR debriefs resolved coding discrepancies and informed minor codebook revisions. The final codebook is available in Supplementary Material S3.

Data from the FADFED, FGDs, and KIIs was synthesized after coding. Analytic team members constructed thick descriptions of relevant social norms codes using all three data sources, noting key similarities and differences across data sources and participant characteristics (gender and campus role) and documenting common intersections with other codes. Team discussions informed the extraction of themes from the intersections identified in the thick descriptions to delineate five major distal norms and their relationship to proximal norms governing perpetration and reporting of SH on campus.

## Results

SH at the university occurred within a complex, and sometimes contradictory, normative environment at the intersection of gendered, tribal, and institutional power. While participants almost universally characterized SH as unacceptable, with FADFED responses describing it is “immature,” “immoral,” “backwards,” “vain,” “disrespectful,” and “dysfunctional,” almost all student participants conceded it was common on campus and in Jordanian society more broadly. Norms proscribing harassment were perceived as weakly enforced, primarily because negative sanctioning of harassers was unlikely.

**FIGURE 1:**
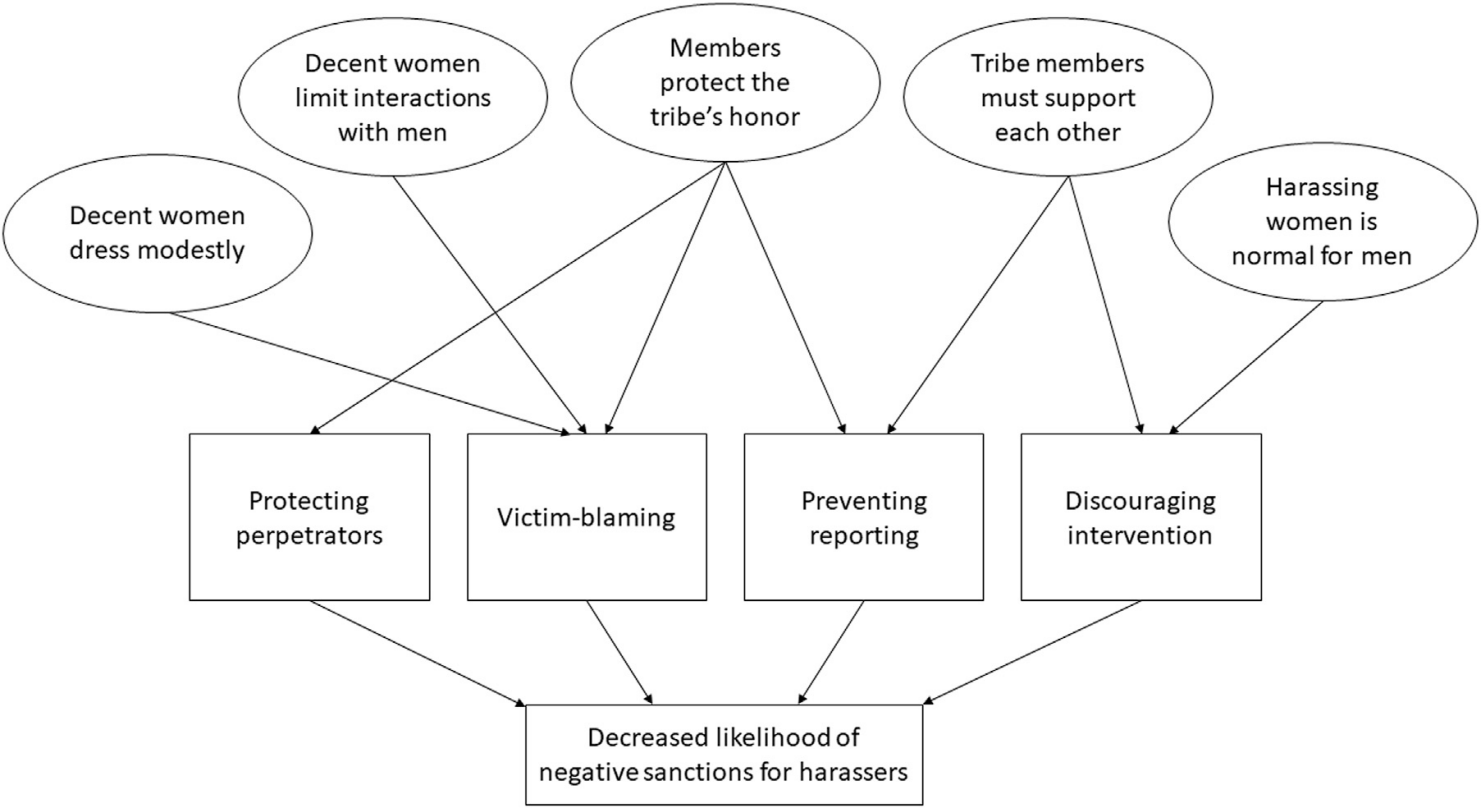
Distal norms contributing to decreased sanctions against harassers.

Distal norms related to gender and tribal affiliation served to weaken norms against SH further by 1) protecting the perpetrator, 2) preventing reporting, 3) blaming the victim, and/or 4) discouraging bystander intervention. Table 2 describes these avenues of influence in more detail and provides exemplary quotes for the relationships depicted in Figure 1.

**TABLE 2:** Distal norms influencing SH and their perceived impact on SH behavior.

	Normative expectations	Avenue of influence on SH behavior	Example
Expectations of appropriate behavior for men and women (gender norms)	Decent women dress modestly	Decreases likelihood of negative sanctions by placing blame on the victim	“When a girl isn’t covering her hair and wearing tight clothes, or something, the guy cannot take the entire blame.” (male FGD)
	Decent women limit interactions with men	Decreases likelihood of negative sanctions by placing blame on the victim	“This is only in our university, especially the faculty of languages, it’s another world. You are not even in Jordan. You find a girl lying down smoking, a guy sleeping on a girl’s lap, that’s very normal.” (female FGD)
	Harassing women is normal for men	Decreases bystander intervention by male peers by normalizing SH and linking SH to masculinity	“Any guy who treats a lady with respect, what do they call him? A sheep.” (female FGD)
Expectations of appropriate behavior for tribe members	Tribe members must support each other	Decreases likelihood of negative sanctions by obligating tribe members defend each other against accusations of SH	“If he (a perpetrator of SH) has a *wasta*, he (a university official) will make sure nothing happens to him.” (female FGD)
	Members protect the tribe’s honor	Increases likelihood of negative sanctions (for out-group harassers) by obligating male tribe members to defend female tribe members who are harassed	“Cases that the Jordanian society knew about were caused by a male student who harassed a female student by saying an inappropriate word, so her cousins and his cousins gathered and had a fight. Lots of fights on campus are a results of sexual harassment.” (administrator)
		Decreases likelihood of sanctions and detectability by making women afraid to report harassment	“Her family will also take a wrong idea about her, they will keep her under the radar because they will start thinking that she must’ve done something wrong. They wouldn’t assume that the boy is a psychopath, no, she must’ve seduced him.” (female FGD)

In some cases, the detectability of the SH behavior also played a role. Many students, for example, mentioned instances of sexual coercion by professors as occurring in private spaces, usually the professor’s office after class. However, the majority of harassment was believed to occur in public spaces in the form of staring, verbal comments, and occasionally, physical touching. These public forms of harassment were almost often described as crimes of opportunity perpetrated by strangers, limiting victim’s ability to report the incident and thereby further reducing the likelihood of sanctions.

### *They’ll Give the Professor 100 Excuses*: Harassers are Protected by Their Peers

A major weakness in proximal norms proscribing SH behaviors emerging in student narratives was harassers’ perceived immunity to negative sanctions. Members of powerful tribes and individuals with institutional power, such as faculty members and security personnel, were described as particularly unlikely to suffer any consequences for harassing women. In a focus group discussion, one woman discussed the dilemma that a female student faced when a professor harassed her:

She thought, “If I tell the dean, nothing will happen.” She lost hope and was afraid because the first thing she was going to hear is, “What took you to the professor’s office? Just like any other student, you could’ve waited in the class to ask him about anything or to take anything from him, or you could’ve asked the rest of the students.” They’ll give the professor 100 excuses.

In particular, norms governing intratribal relationships and family honor appeared in many student narratives and often manifested in gendered ways. For men, mutual obligations to support one’s own tribe could take the form of publicly defending a tribe member accused of SH, despite their personal feelings. Male students in FGDs discussed how these expectations would govern men’s public response to a fellow tribe member being accused, and how this immunity from negative sanctions could encourage future harassment:

Participant 1: If you know that someone has your back, and that you won’t be held accountable no matter what, why don’t you just act as you please?

Facilitator: You’re saying that if a member of a tribe makes a mistake, the other members will support him?

Participant 1: Yes, they will.

Participant 2: I don’t want to say “support him”, but at least they will turn a blind eye.

Facilitator: So they don’t try to correct his behavior?

Partcipant 2: They just turn a blind eye … they may talk about it internally, but they have to defend him in public.

Participant 1: They stand by his side.

Male students relayed that even informal sanctioning of fellow tribe members who perpetrated harassment was rare. These obligations were perceived to supersede official policy and procedure when cases were reported formally to university administration, as related by a female FGD participant:

Even if she files a complaint, nothing will happen. Because most of them have connections and will keep things quiet. She won’t gain anything, on the contrary, she’ll be devastated.

However, informal sanctioning of out-group harassers of in-group women to protect the tribe’s honor was perceived as more common. Faculty, staff, and students agreed that harassment of a female tribe member could result in violent reprisal from the woman’s male relatives and identified SH as an instigating factor in violence occurring on campus. One campus administrator noted that such informal support channels also might limit reporting of SH through formal complaint structures.

I’m sure there are cases that we do not know about because the student decided not to speak out … many times a girl or a guy would not come to us and instead they seek help from their father or family/tribe.

### *She Will Bring a Scandal to the Family*: Reporting Harassment has Consequences for Women and Their Relatives

Men’s normative expectations of family and tribal support manifested not only through protection of in-group perpetrators, but also through protection and control of female relatives as a means of defending family and tribal honor. This intersection of gendered and tribal normative expectations created major barriers to reporting for women: disclosure of SH victimization to family would carry the risk of causing a “scandal,” since family honor was tightly bound to daughters’ behavior and perceived decency. While family members were perceived as more likely to sanction harassers outside of the tribe, the consequences of disclosing SH for women ranged from restrictions on their mobility to honor killings. Women in an FGD explained that they would not disclose an incident of SH even to “open-minded” family members due to concerns of being more closely monitored by their relatives:

Participant 1: For example, I haven’t told my family the story of (being groped on) the bus, ask me why? Because they won’t understand, they won’t get me a car (laughs).

Participant 2: They won’t understand they will get worried about you every time you leave the house.

Participant 3: Yeah, they will start ringing me every time I leave ... we live in an Eastern society, even though my parents are open minded, but still they would worry about me as a girl.

Likewise, a faculty administrator related a story highlighting the challenges that considerations of family honor posed to formal reporting to university administration, implicating family members in discouraging formal help-seeking:

Her family told her that she will bring a scandal to the family and that nobody would believe that she is an innocent and that a faculty member is the one who harassed her. This is a case that I have experienced, and although we have provided all the support and assured her that nothing will happen to her, but she still withdrew the complaint. I cannot do anything when a student cancels a complaint, so this is the difficulty that is on the female students who are afraid and consider the social pressure or the benefits like when I told you that she was afraid of failing the class. And many times, the family is the main reason that prevents them from filing a complaints, and what’s more dangerous/concerning is that they seek to solve these issues outside the border of the university, on a personal level.

Thus, norms governing family and tribal honor generally served to protect harassers from negative sanctions in two ways: supporting in-group harassers against accusations and preventing women from reporting harassment generally.

### *The Guy Cannot Take the Entire Blame*: Victim-Blaming is Used to Justify Harassment

The emphasis on women’s behavior as a reflection upon her entire family was tightly connected with strict gender norms within a patriarchal society. One of the more common ways by which young men were able to circumvent the unacceptability of SH was blaming the victim for “provoking” the harassment by violating norms of feminine “decency.” Norms governing women’s behavior emerged in both men’s and women’s narratives as a means of justifying SH and/or framing the behavior as harmless. Women were expected to dress modestly, which could entail anything from wearing *hijab* (head scarf) and *jilbab* (loose, ankle-length garment) to simply avoiding tight and revealing clothing, and to limit interactions with men. While these expectations also fell on men to a lesser degree, adherence to gender norms distinguished “decent” women from acceptable targets of SH, who were often perceived to have invited the behavior. Below, a male student discussed his criteria for assessing whether it was acceptable to stare at a female student:

When a girl is covering her hair or wearing modest clothes, I may look at her, but when a girl isn’t covering her hair and wearing tight clothes, the guy cannot take the entire blame … I find a girl, to be honest, wearing something too revealing, too attractive, of course, I will look at her, what do you expect me to do? Ok, I am not going to harass her, but I will have to look, but if I see a girl covering her hair and wearing a long dress, I don’t look.

Such judgements were by no means limited to male students, with many female students also implicating women’s dress and behavior as causes of harassment.

While some students viewed adherence to gender norms as protective, many maintained that strict adherence might lessen the degree of harassment but not the overall risk, and still others maintained that standards of female “decency” were merely a post-hoc justification used by harassers. The importance of adherence to standards of dress and behavior was a subject of intense disagreement in both men’s and women’s focus groups, a disagreement that perhaps reflected the broader range of normative expectations and attitudes represented on campus. Student focus groups and interviews with staff commonly implicated such differences in normative expectations of behavior as a major cause of SH.

**FIGURE 2:**
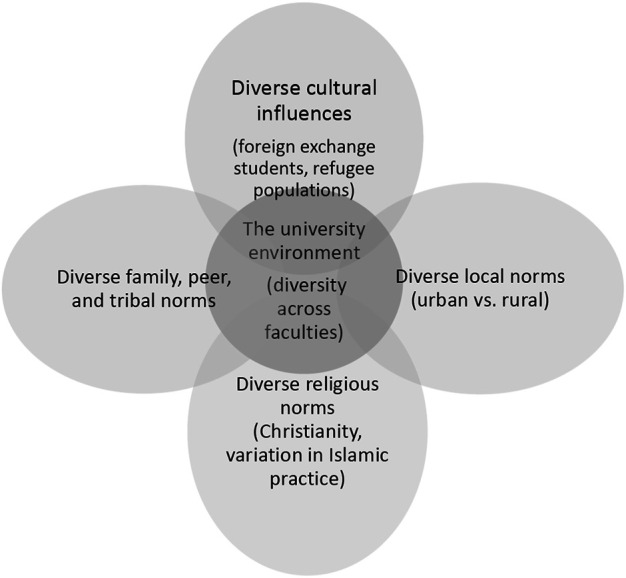
Factors influencing the diversity of campus norms.

With students from across Jordan, the Arab region, and the world, the campus environment could be interpreted as a nested normative environment in which many behaviors considered inappropriate within public spaces were perceived to occur (Figure 2). Students’ narratives featured mention of tight and revealing clothing worn by some female students and faculty and physical contact between male and female students as examples of the exceptionality of the campus environment. There was consensus across FGDs and KIIs that norms varied across faculties as well, with humanities and arts mentioned as the most permissive areas of campus and other areas described as more conservative. A female student expressed frustration with this permissiveness in one FGD:

We have reached a point where we are not held accountable for what we wear … (this university) is one of the top, it is very open to different cultures and we have a lot of exchange students. We can’t force exchange students to wear a long dress and veil, that would be wrong. But what happens is when I am with my girl friends I hear them say, “Look what she is wearing!”

At the same time, since students came from all over the country, there also were many who might subscribe to more conservative norms than those who grew up in urban areas such as those surrounding the campus. Many students identified the wide variation in norms within the campus environment as a factor that exacerbated SH. A male student described the normative conflict experienced by students from more conservative parts of Jordan in mixed-gender settings for the first time:

…they always see even their aunts fully covered, so they come here … and they see girls wearing trousers and shirts. The girls think they’re just being themselves, and their families don’t mind when they see them leaving their homes, but then these guys come here, and they just can’t help but talk about her, and harass her.

Students originating from rural areas, in particular, were cited by both students and staff as likely to judge almost any interaction between unrelated men and women as unseemly. For example, a key staff member involved in campus security described normative expectations among rural students as prohibiting even greeting:

There is a difference between (a woman) who lives in a main city and understands the community well and has interacted with boys since first or sixth grade until high school and that’s her culture, and when she gets into college/university this is her culture … in the street, if I saw you and said “hi” you would say “hi,” but if I said this to another girl, she might look on the other side, and if I repeated this she might ask what’s wrong with me, what do you want, do you know me well?

Some students of both genders also perceived that more conservative gender norms reserved the public arena as a male space, and that women were violating this norm even by going to university. Participants viewed these norms as prevalent among students who grew up outside the city and were accustomed to single-gender education and socialization.

### *Friends are the Most Influential*: Male Peers Normalize SH and Discourage Intervention

The diversity of opinion on the influence of gender norms on SH was mirrored in the conflicting normative expectations around the acceptability of SH that emerged in all three men’s focus groups. While FADFED data suggested that SH was universally considered unacceptable according to Jordanian norms, FGDs presented a more nuanced picture, where SH could be viewed as acceptable, and even expected, depending on the perceived severity of the behavior, the perceived decency of the target, and the audience witnessing the act. Milder SH behaviors, such as staring and verbal comments, were characterized in men’s FGDs as a normal, harmless way to pass time and bond with peers. Such behavior was normalized to such a degree that men who intervened to prevent peers from engaging in SH could expect to be mocked and socially excluded. Male students discussed the social consequences of standing up to their friends and refusing to harass women:

Facilitator: One or two of the boys is fed up with this kind of behavior and tells his friends to stop being jerks. What do you think will happen?

Participant 1: They’ll make fun of him.

Participant 2: They’ll tell him, “Why are you acting like a saint?”

Facilitator: Would his friends listen to him or would they ignore his rebuke?

Participant 3: They would ignore him.

Participant 4: They would ignore him and stop hanging out with him.

Female students were aware of social pressure experienced by their male counterparts who might want to speak out against SH, noting that respecting women could result in questions about one’s masculinity:

There will be sarcasm and ridicule on the guy ... they will tell him you are not a man... they will say you are a “sheep”, that’s the term that they use. Any guy who treats a lady with respect, they call him what-a sheep.

While male students cited “lowering the gaze” as a moral norm dictated in the Quran, many of the same men admitted that they often failed to adhere to it. When spending time with other young men on campus, the expectations of peer reference groups tended to be more salient than those of family members or religious leaders who might consider SH to be immoral and unacceptable. Male FGD participants described this distinction between men’s behavior for authority figures versus peers:

Nobody is going to harass in front of his father or mother, but friends are the most influential, in front of your friends, you feel comfortable and will act yourself, you don’t have anyone to watch you, if they want to guide you to the right path, they will tell you that this is wrong, but mostly, this doesn’t happen, and you can harass freely.

## Discussion

While there was a universal perception among all participant samples that SH was not an acceptable behavior on the university campus or in the broader Jordanian society, its perceived prevalence belied the weakness of the norm in reducing or preventing SH behavior. This weakness was primarily attributed to a lack of sanctions, resulting from gendered distal norms that discouraged reporting and intervening, shifted blame onto victims, and protected perpetrators through tribal connections. In some cases, milder forms of SH were also normalized by men’s peer groups.

Young men in our sample described certain SH behaviors, such as staring and verbal commenting, as “normal” and inevitable, citing justifications such as the target’s dress or demeanor to characterize harassment as harmless or desired. The finding that SH is more acceptable under certain circumstances aligns with research suggesting that norms that increase the acceptability of violence against women are often mediated by the perceived severity of the violence and the reasons used to justify the violence (Cislaghi and Heise, 2016). Addressing the impact of SH on victims through open dialogue and publicizing men’s private opinions on the acceptability of harassment may be a promising approach toward reducing the perception of “acceptable SH” among men, and thereby reducing barriers to bystander intervention. Prior studies among university men in the United States have demonstrated the efficacy of such approaches in correcting men’s misperceptions and increasing bystander intervention against sexual assault on college campuses (Fabiano et al., 2003). Another notable success in raising awareness about the harms of sexual violence is the documentary film *The Hunting Ground*, which featured personal stories of student survivors and served as a catalyst for student activism in North America, Australia, and the United Kingdom (G. Towl, 2016).

Young men also may face normative pressure from peers to harass women on campus and fear sanctions for opposing this behavior. Social marketing to replace harmful norms that link SH to masculinity must concurrently aim to make intervening to stop harassment more acceptable for men. Creating spaces for young men to reinforce positive masculine norms is another approach that has shown promise in other contexts (WHO, 2009) and that lends itself well to a campus-based approach. Such “norms incubators” are key to the success of diffusion-based social norms change interventions (Clark et al., 2020), whereby a new, positive norm, is practiced in an environment with less risk of negative sanctions. *GlobalConsent*, a web-based program designed to reduce sexual violence and increase bystander behavior among first-year college men in Vietnam, is another intervention that has explicitly challenged harmful norms of masculinity as part of its programming (Kathryn M Yount et al., 2020).

Mirroring findings from various contexts around the world (Henry, 2017; Nahar et al., 2013; Zietz and Das, 2018), gender norms governing appropriate feminine behavior also featured prominently in participant narratives, and women’s perceived violation of gender norms was cited as a major justification for SH. Although individuals perceived to violate gender norms are also at increased risk of SH at universities in the United States and Europe (Dall’Ara and Maass, 1999; Klein and Martin, 2019; National Academies of Sciences, E., & Medicine, 2018), inequitable gender norms are rarely highlighted in campus-based prevention programs in these Global North settings (G. J. Towl and Walker, 2019), with some notable exceptions (Salazar et al., 2019). HarassMap, an Egyptian anti-harassment activist group, has effectively used mass marketing campaigns such as “Why Does He Harass” and “Get It Right” to address the gendered normative expectations that contribute to men’s justification of harassment in Egypt (Cochrane et al., 2019).

Breaking down normative barriers to reporting is perhaps the most complex challenge in combatting SH on the university campus. Norms governing the behavior of tribe members emerged prominently as a barrier to sanctioning and reporting SH, both formally and informally. The salience of formal sanctions against SH can be addressed primarily through strengthening official policies and procedures at the university, and there is no shortage of policy recommendations developed to assist universities in tackling SH (National Association of Student Personnel Administrators, 2017) and making reporting the “new norm” (G. J. Towl and Walker, 2019). In the Arab region, Egypt’s HarassMap has engaged universities and other organizations in creating designated units to receive and deal with SH complaints (Cochrane et al., 2019).

However, in the Jordanian context, policy change alone may be insufficient while norms governing family and tribal honor persist in discouraging women from “causing a scandal” by reporting SH and by encouraging tribe members to “turn a blind eye” when members harass. To address the former, engaging family members through open days for prospective students may be one way to assure students and their parents that reports will remain confidential and that those who report will be protected (G. J. Towl and Walker, 2019). Student and faculty participants noted that in Jordan, informal tribal networks often exerted influence on university structures and that informal sanctions through these networks might actually be more likely to occur in the Jordanian context. Developing social norms programming that leverages students’ family and tribal networks for diffusion of positive norms and emphasizes the harm to the tribe when a member harasses women (for example, by increasing the risk of intertribal conflict) might encourage men to sanction peers who perpetrate SH, at least privately. Because crimes related to honor and morality have traditionally fallen under the jurisdiction of tribal leadership (Sonbol, 2003), this may be an avenue to increase the strength and likelihood of sanctions in a way that is not a violation of existing cultural/legal norms.

### Strengths and Limitations

As is the case with all qualitative research, our study is not intended to be representative of university campuses in Jordan or elsewhere, but to provide in-depth, nuanced picture of the normative climate around SH at one university. A notable strength of our approach is the use of multiple, complementary samples and data collection methods to triangulate and validate our findings. Despite our efforts to achieve a diverse sample, we were unable to interview any female staff or faculty as key informants due to our eligibility criteria of having been involved with cases of SH, omitting potentially insightful perspectives. Indeed, the lack of female personnel involved in SH response on campus was, in and of itself, an important finding, and one that was explicitly addressed by one key informant. Secondly, data was collected in Arabic and translated into English for interpretation, creating the potential for loss of nuance and context. To mitigate this, analytic team members, the majority of whom were English-Arabic bilingual, often returned to the original Arabic transcripts to validate findings and clarify phrasing.

## Conclusion

Sexual harassment at the university was regarded as unacceptable, yet the behavior persisted due to the unlikelihood of sanctions, which were primarily the result of stronger distal norms related to gender and tribal affiliation. The complexity of the normative environment surrounding SH perpetration will necessitate the use of targeted, parallel approaches to strengthen proximal norms proscribing SH and to change distal norms supporting SH and discouraging reporting. Strengthening weak norms against SH will require increasing the likelihood of sanctions, whether formally (by revising university policies and procedures to increase accountability) or informally (by increasing the acceptability of bystander intervention and fostering tribal investment in sanctioning members who harass women). Creating dialogue that emphasizes the harmful nature of SH behaviors that young men often perceive as harmless, such as staring or verbal comments, may also be an effective strategy to shift norms that govern how male students interact in the presence of peers. Any social norms change intervention will need to consider the various reference groups that dictate and enforce norms surrounding SH within the context of a patriarchal society.

## Data Availability

The raw data supporting the conclusions of this article will be made available by the authors, without undue reservation.

## Footnotes

1
*Wasta* (Jordanian Arabic) refers to a powerful social connection.

## References

[B1] Biernacki P. Waldorf D. (1981). Snowball Sampling: Problems and Techniques of Chain Referral Sampling. Sociol. Methods Res. 10 (2), 141–163. 10.1177/004912418101000205

[B2] Bondestam F. Lundqvist M. (2020). Sexual Harassment in Higher Education - a Systematic Review. Eur. J. Higher Educ. 10 (4), 397–419. 10.1080/21568235.2020.1729833

[B3] Chafai H. (2017). Contextualising Street Sexual Harassment in Morocco: a Discriminatory Sociocultural Representation of Women. J. North Afr. Stud. 22 (5), 821–840. 10.1080/13629387.2017.1364633

[B4] Cislaghi B. Heise L. C. (2016). Measuring Gender-Related Social Norms: Report of a Meeting. Baltimore, Maryland. *June 14-15, 2016*. Retrieved from Lonson.

[B5] Cislaghi B. Heise L. (2018a). Four Avenues of Normative Influence: A Research Agenda for Health Promotion in Low and Mid-income Countries. Health Psychol. 37 (6), 562–573. 10.1037/hea0000618 29672098

[B6] Cislaghi B. Heise L. (2018b). Theory and Practice of Social Norms Interventions: Eight Common Pitfalls. Global. health 14 (1), 1–10. 10.1186/s12992-018-0398-x 30119638 PMC6098623

[B7] Cislaghi B. Heise L. (2020). Gender Norms and Social Norms: Differences, Similarities and Why They Matter in Prevention Science. Sociol. Health Illn 42 (2), 407–422. 10.1111/1467-9566.13008 31833073 PMC7028109

[B8] Clark C. J. Ferguson G. Shrestha B. Shrestha P. N. Oakes J. M. Gupta J. (2018). Social Norms and Women's Risk of Intimate Partner Violence in Nepal. Soc. Sci. Med. 202, 162–169. 10.1016/j.socscimed.2018.02.017 29549822

[B9] Clark C. J. Shrestha B. Ferguson G. Shrestha P. N. Calvert C. Gupta J. (2020). Impact of the Change Starts at Home Trial on Women's Experience of Intimate Partner Violence in Nepal. SSM - Popul. Health 10, 100530. 10.1016/j.ssmph.2019.100530 31890850 PMC6928358

[B64] Cislaghi B. Heise L. C. (2016). Measuring Gender-related Social Norms. Technical LSHTM, London. Retrieved from: https://researchonline.lshtm.ac.uk/id/eprint/4646972

[B10] Clarke A. E. (2003). Situational Analyses: Grounded Theory Mapping after the Postmodern Turn. Symbolic Interact. 26 (4), 553–576. 10.1525/si.2003.26.4.553

[B11] Cochrane L. Zeid Y. Sharif R. (2019). Mapping Anti-Sexual Harassment and Changing Social Norms in Egypt. ACME: Int. J. Crit. Geographies 18 (2), 394–420.

[B12] Costenbader E. Cislaghi B. Clark C. J. Hinson L. Lenzi R. McCarraher D. R. (2019). Social Norms Measurement: Catching up with Programs and Moving the Field Forward. J. Adolesc. Health 64 (4), S4–S6. 10.1016/j.jadohealth.2019.01.001 30914167 PMC6426726

[B13] Dall'Ara E. Maass A. (1999). Studying Sexual Harassment in the Laboratory: Are Egalitarian Women at Higher Risk?. Sex Roles 41 (9), 681–704. 10.1023/a:1018816025988

[B14] de Lijster G. P. A. Felten H. Kok G. Kocken P. L. (2016). Effects of an Interactive School-Based Program for Preventing Adolescent Sexual Harassment: a Cluster-Randomized Controlled Evaluation Study. J. Youth Adolescence 45 (5), 874–886. 10.1007/s10964-016-0471-9 PMC4826426 27044017

[B15] Fabiano P. M. Perkins H. W. Berkowitz A. Linkenbach J. Stark C. (2003). Engaging Men as Social justice Allies in Ending Violence against Women: Evidence for a Social Norms Approach. J. Am. Coll. Health 52 (3), 105–112. 10.1080/07448480309595732 14992295

[B16] Fan J. Voûte A. Fan J. (2018). Why Some Men Street Harass: Findings, Insights and Solutions from Men in New York City. New York: Street Action For Equality and Respect.

[B17] Fitzgerald L. F. Gelfand M. J. Drasgow F. (1995). Measuring Sexual Harassment: Theoretical and Psychometric Advances. Basic Appl. Soc. Psychol. 17 (4), 425–445. 10.1207/s15324834basp1704_2

[B18] Fitzgerald L. F. Drasgow F. Hulin C. L. Gelfand M. J. Magley V. J. (1997). Antecedents and Consequences of Sexual Harassment in Organizations: a Test of an Integrated Model. J. Appl. Psychol. 82 (4), 578–589. 10.1037/0021-9010.82.4.578 9378685

[B19] Galdi S. Maass A. Cadinu M. (2014). Objectifying Media. Psychol. Women Q. 38 (3), 398–413. 10.1177/0361684313515185

[B20] Glaser B. G. Strauss A. L. (2017). Discovery of Grounded Theory: Strategies for Qualitative Research. New York: Routledge. 10.4324/9780203793206

[B21] Hardies K. (2019). Personality, Social Norms, and Sexual Harassment in the Workplace. Personal. Indiv. Differ. 151, 109496. 10.1016/j.paid.2019.07.006

[B22] Hartmann M. Krishnan S. (2016). Ethical and Safety Recommendations for Intervention Research on Violence against Women. Building on Lessons from the WHO Publication. Geneva: World Health Organization Retrieved from: https://apps.who.int/iris/bitstream/handle/10665/251759/9789241510189-eng.pdf

[B23] Hayford S. R. Garver S. Soura A. B. Cheong Y. F. Grose R. G. Yount K. M. (2020). Community Influences on Female Genital Mutilation/cutting: A Comparison of Four Francophone West African Countries. Stud. Fam. Plann. 51 (1), 3–32. 10.1111/sifp.12109 32103517 PMC12703717

[B24] Heilman B. Barker G. (2018). Unmasking Sexual Harassment—How Toxic Masculinities Drive Men’s Abuse in the US, UK, and Mexico and what We Can Do to End it. Washington, D.C.: Promundo-US.

[B25] Hennink M. Hutter I. Bailey A. (2020). Qualitative Research Methods. London: Sage.

[B26] Henry H. M. (2017). Sexual Harassment in the Egyptian Streets: Feminist Theory Revisited. Sex. Cult. 21 (1), 270–286. 10.1007/s12119-016-9393-7

[B27] Huerta M. Cortina L. M. Pang J. S. Torges C. M. Magley V. J. (2006). Sex and Power in the Academy: Modeling Sexual Harassment in the Lives of College Women. Pers Soc. Psychol. Bull. 32 (5), 616–628. 10.1177/0146167205284281 16702155

[B28] Insight I. (2010). The Dynamics of Social Change towards the Abandonment of Female Genital Mutilation/cutting in Five African Countries. New York: UNICEF.

[B29] Jewell J. Spears Brown C. Perry B. (2015). All My Friends Are Doing it: Potentially Offensive Sexual Behavior Perpetration within Adolescent Social Networks. J. Res. Adolesc. 25 (3), 592–604. 10.1111/jora.12150

[B30] Jewkes R. Flood M. Lang J. (2015). From Work with Men and Boys to Changes of Social Norms and Reduction of Inequities in Gender Relations: a Conceptual Shift in Prevention of Violence against Women and Girls. Lancet 385 (9977), 1580–1589. 10.1016/s0140-6736(14)61683-4 25467578

[B31] Jordan population and family health survey 2012. (2013). Retrieved from Department of Statistics/Jordan and ICF International.

[B32] Karim N. Greene M. Picard M. (2016). The Cultural Context of Child Marriage in Nepal and Bangladesh: Findings from CARE's Tipping Point Project Community Participatory Analysis: Research Report. Geneva. CARE.

[B33] Klein L. Martin S. L. (2019). Sexual Harassment of College and university Students: a Systematic Review. Trauma. Violence. Abuse, 1524838019881731. 10.1177/1524838019881731 31635552

[B34] Kyegombe N. Starmann E. Devries K. M. Michau L. Nakuti J. Musuya T. (2014). 'SASA! Is the Medicine that Treats Violence'. Qualitative Findings on How a Community Mobilisation Intervention to Prevent Violence against Women Created Change in Kampala, Uganda. Glob. Health Action. 7 (1), 25082. 10.3402/gha.v7.25082 25226421 PMC4165071

[B35] Lindsey U. (2012). Arab Women Make Inroads in Higher Education but Often Find Dead Ends. Washigton, D.C.: Chronicle of Higher Education.

[B36] Mackie G. Moneti F. Shakya H. Denny E. (2015). What Are Social Norms? How Are They Measured. San Diego: University of California at San Diego-UNICEF Working Paper.

[B37] McHugh M. L. (2012). Interrater Reliability: The Kappa Statistic. Biochem. Med. 22 (3), 276–282. 10.11613/bm.2012.031 PMC3900052 23092060

[B38] McLaughlin H. Uggen C. Blackstone A. (2017). The Economic and Career Effects of Sexual Harassment on Working Women. Gend. Soc. 31 (3), 333–358. 10.1177/0891243217704631 29056822 PMC5644356

[B39] Muazzam A. Qayyum F. Cheng J. (2016). Experiences of Sexual Harassment: Interplay of Working Environment, Depression and Self-Esteem in Pakistani Women. Pakistan J. Soc. Clin. Psychol. 14 (1), 42.

[B40] Murdoch M. Pryor J. B. Anderson Polusny M. Gackstetter G. D. Cowper Ripley D. (2009). Local Social Norms and Military Sexual Stressors: Do Senior Officers' Norms Matter? Mil. Med. 174 (10), 1100–1104. 10.7205/milmed-d-04-2308 19891224 PMC6553466

[B41] Nahar P. Van Reeuwijk M. Reis R. (2013). Contextualising Sexual Harassment of Adolescent Girls in Bangladesh. Reprod. Health Matters 21 (41), 78–86. 10.1016/s0968-8080(13)41696-8 23684190

[B42] National Academies of Sciences, E., & Medicine (2018). Sexual Harassment of Women: Climate, Culture, and Consequences in Academic Sciences, Engineering, and Medicine. Washington, D.C.: National Academies Press. 29894119

[B43] National Association of Student Personnel Administrators (2017). Culture of Respect CORE Evaluation. Retrieved from Washington, D.C.

[B44] Paluck E. L. Ball L. Poynton C. Sieloff S. (2010). Social Norms Marketing Aimed at Gender Based Violence: A Literature Review and Critical Assessment. New York: International Rescue Committee.

[B45] Pina A. Gannon T. Saunders B. (2009). An Overview of the Literature on Sexual Harassment: Perpetrator, Theory, and Treatment Issues. Aggression Violent Behav . 14, 126–138. 10.1016/j.avb.2009.01.002

[B46] Pinchevsky G. M. Magnuson A. B. Augustyn M. B. Rennison C. M. (2019). Sexual Victimization and Sexual Harassment Among College Students: a Comparative Analysis. J. Fam. Violence, 35, 603–618. 10.1007/s10896-019-00082-y

[B47] Pryor J. B. LaVite C. M. Stoller L. M. (1993). A Social Psychological Analysis of Sexual Harassment: The Person/situation Interaction. J. vocational Behav. 42 (1), 68–83. 10.1006/jvbe.1993.1005

[B48] Salazar L. F. Vivolo-Kantor A. Schipani-McLaughlin A. M. (2019). Theoretical Mediators of RealConsent: a Web-Based Sexual Violence Prevention and Bystander Education Program. Health Educ. Behav. 46 (1), 79–88. 10.1177/1090198118779126 29996689

[B49] Smith J. C. Jr (1980). Prologue to the EEOC Guidelines on Sexual Harassment. Baltimore: University of Baltimore Law Review. *Cap* 10 UL Rev., 471.

[B50] Software V. (2019). MAXQDA 2018. Retrieved from: https://www.maxqda.com/help-max18/welcome.

[B51] Sonbol A. (2003). Women of the Jordan: Islam, Labor, and the Law. Syracuse: Syracuse University Press.

[B52] Tenbrunsel A. E. Rees M. R. Diekmann K. A. (2019). Sexual Harassment in Academia: Ethical Climates and Bounded Ethicality. Annu. Rev. Psychol. 70, 245–270. 10.1146/annurev-psych-010418-102945 30156976

[B53] Towl G. J. Walker T. (2019). Tackling Sexual Violence at Universities: An International Perspective. London: Routledge. 10.4324/9781351201995

[B54] Towl G. (2016). Tackling Sexual Violence at UK Universities: a Case Study. Contemp. Soc. Sci. 11 (4), 432–437. 10.1080/21582041.2016.1260764

[B55] Tyler A. Boxer D. (1996). Sexual Harassment? Cross-Cultural/cross-Linguistic Perspectives. Discourse Soc. 7 (1), 107–133. 10.1177/0957926596007001005

[B56] Whaley G. L. Tucker S. H. (1998). A Theoretical Integration of Sexual Harassment Models. Equal Opportunities Int. 17 (1), 21–29. 10.1108/02610159810785485

[B57] What is FADFED? (2013). Retrieved from: www.fadfed.org.

[B58] WHO (2009). Changing Cultural and Social Norms that Support Violence. Geneva: World Health Organization. (9241598336). Retrieved from. 10.2471/tdr.09.978-924-1598767

[B59] Willness C. R. Steel P. Lee K. (2007). A Meta-Analysis of the Antecedents and Consequences of Workplace Sexual Harassment. Personnel Psychol. 60 (1), 127–162. 10.1111/j.1744-6570.2007.00067.x

[B60] Yount K. M. Minh T. H. Trang Q. T. Cheong Y. F. Bergenfeld I. Sales J. M. J. B. p. h. (2020). Preventing Sexual Violence in College Men: A Randomized-Controlled Trial of GlobalConsent. 20(1), 1–19. 10.1186/s12889-020-09454-2 PMC7466489 32873262

[B61] Yount K. M. Clark C. J. Bergenfeld I. Khan Z. Cheong Y. F. Kalra S. (XXX). Impact Evaluation of the Care Tipping Point Initiative in Nepal: A Mixed-Methods Cluster-Randomized Controlled Trial. BMJ Open. (under review). 10.1101/2021.07.01.21259594 PMC8314701 34312191

[B62] Zambelli E. Mainardi A. Hajek A. (2018). Sexuality and Power in Contemporary Italy: Subjectivities Between Gender Norms, Agency and Social Transformation. Mod. Italy 23 (2), 129–138. 10.1017/mit.2018.11

[B63] Zietz S. Das M. (2018). ‘Nobody Teases Good Girls': A Qualitative Study on Perceptions of Sexual Harassment Among Young Men in a Slum of Mumbai. Glob. Public Health 13 (9), 1229–1240. 10.1080/17441692.2017.1335337 28580845 PMC6690339

