# Supplementary material for: Diagnosing Norms Surrounding Sexual Harassment at a Jordanian University
Source: Front Sociol. 2021 Jul 26;6:667220. doi: 10.3389/fsoc.2021.667220 (PMC8350132; doi:10.3389/fsoc.2021.667220)
Supplement: Supplementary file 1 [file Table1.DOCX]

**Participants Needed in a Research Study:**

**Gender-based Violence and Sexual Harassment Among Jordanian University Students**


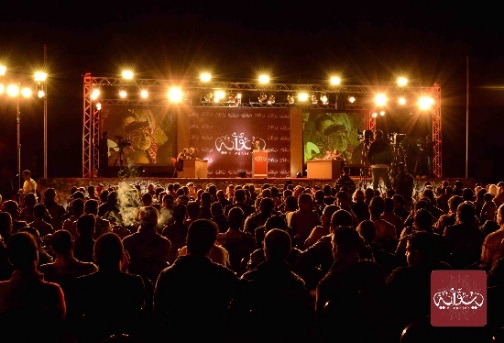


We are seeking current University of Jordan freshmen and sophomore students 18 and over to participate in focus group discussions regarding gender-based violence and sexual harassment.

Participation involves speaking with a group of fellow students for about an hour. Participants will receive 5 JOD for their participation.

Please contact Dr. Abeer Dababneh, CWS-UJ Director, at 06 535 5000 or [abeer.dababneh75@gmail.com](mailto:abeer.dababneh75@gmail.com) for more information.

Our study seeks to enhance and evaluate a **locally-based, multi-level intervention** to prevent gender-based violence and sexual harassment on the University of Jordan Campus.


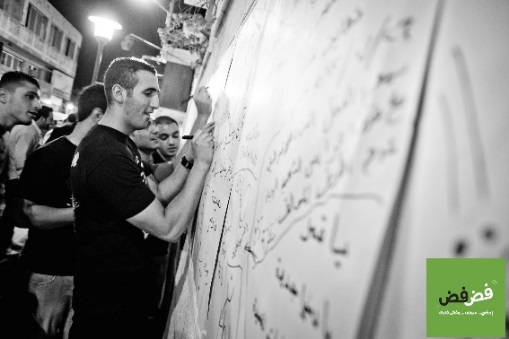


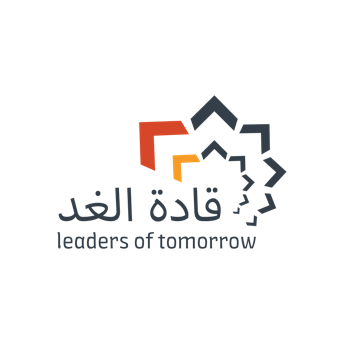


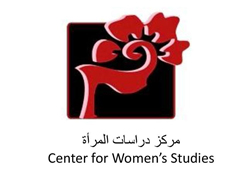

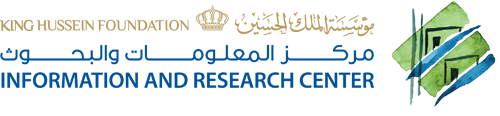

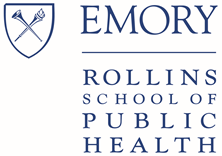


**Sexual Harassment Among Jordanian University Students: Pilot Test of a Promising Primary Prevention Intervention**


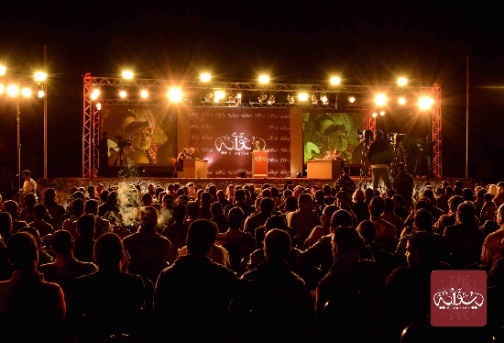


**Objective**

Our study seeks to enhance and evaluate a **locally-based, multi-level intervention** to prevent sexual harassment (SH) on the University of Jordan Campus.

**Our Intervention**


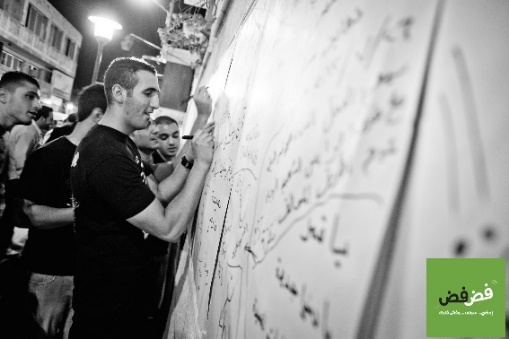
Our multi-level intervention consists of one, three-hour long public debate conducted on campus (Diwanieh), one, 48-hour intensive training for student change makers (Mo7aka), a four-month long social media marketing component conducted online (#ihtarimni), two, three-hour long training sessions for staff, and the establishment of a university steering committee to oversee the project and boost eventual institutionalization of successful components of the project. These activities seek to address key risk factors for SH perpetration including: knowledge of SH, skills to prevent SH, perceptions of social norms that SH is permissible, and the lack of effective SH prevention remedies.^1,38^ We address student, staff, and school level contributors because social and physical contexts are an important factor in the adoption and maintenance of behaviors^69^ and research demonstrates that effective SH interventions are multi-level.^12,34,39^

**Aims**

Our mixed methods study engages students and school leadership to achieve several aims:

1. Using focus group discussions, surveys, and a FADFED (a qualitative data collection tool developed in Jordan), we describe the prevalence, nature, and correlates of SH. We conduct an audit of school SH policies to develop an understanding of the issue of SH from the school perspective
2. Through a coordinated, multi-stakeholder led process, we adapt and enhance an existing intervention and create the first packaged intervention to prevent SH in the Middle Eastern context.

3) Finally, we pilot the intervention and conduct post- tests with participants to examine change over time in SH experiences, perceptions, and school policies among men and women.

**Key Partners**

Team members have a total of 40 years of experience working on and safely researching gender-based violence in the Middle East,^9,70^ including Jordan,^9,71-76^ experience working with youth on sensitive issues, and a proven record of safely researching gender-based violence together in Jordan.^9^ The team consists of the following experts:

**Co-Principal Investigators**

Dr. Aida Essaid, Director, Information and Research Center – King Hussein Foundation (IRCKHF);

Dr. Cari Clark, Associate Professor, Emory University, Rollins School of Public Health;

**Co-Investigators**

Dr. Abeer Dababneh, Director of the University of Jordan’s Center for Women’s Studies (CFWS); Dr. Sami Hourani, Director, Leaders of Tomorrow; Ms. Rachael Spencer, PhD Student, Emory University.

**Future Goals**

We are committed to scaling up and conducting more rigorous evaluations of the intervention. Next steps include:

- Adding a comparison site in Jordan or neighboring country to strengthen the rigor of our study design;
- Increasing research efforts to engage men and boys in understanding ways to prevent SH; and
- Adapting and scaling-up the intervention in university contexts around the region.

**Gender Based Violence Among Jordanian University Students: Pilot Test of a Promising Primary Prevention Intervention Targeting Sexual Harassment**


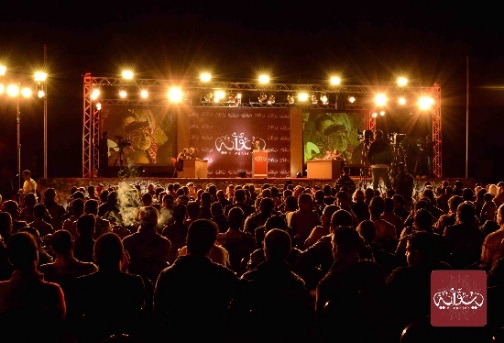


**Objective**

Our study seeks to enhance and evaluate a **locally-based, multi-level intervention** to prevent sexual harassment (SH) on the University of Jordan Campus.

**Our Intervention**


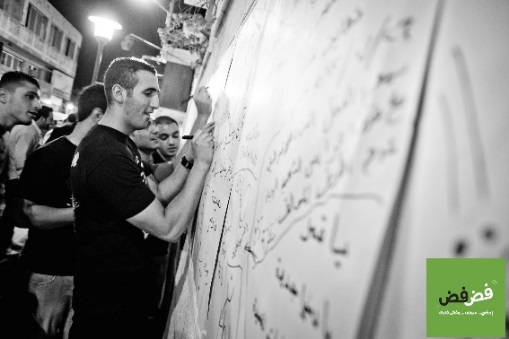
Our multi-level intervention consists of one, three-hour long public debate conducted on campus (Diwanieh), one, 48-hour intensive training for student change makers (Mo7aka), a four-month long social media marketing component conducted online (#ihtarimni), two, three-hour long

training sessions for staff, and the establishment of a university steering committee to oversee the project and boost eventual institutionalization of successful components of the project. These activities seek to address key risk factors for SH perpetration including: knowledge of SH, skills to prevent SH, perceptions of social norms that SH is permissible, and the lack of effective SH prevention remedies. We address student, staff, and school level contributors because social and physical contexts are an important factor in the adoption and maintenance of behaviors and research demonstrates that effective SH interventions are multi-level.

**Aims**

1. The purpose of this study is to evaluate a project to prevent sexual harassment (SH) on campus.
2. The study is funded by a grant from the World Bank and the Sexual Violence Research Initiative.
3. The duration of your participation depends on the activity you participate in.
   1. FADFED: 10 minutes
   2. Focus group discussions: about an hour and a half
   3. Stakeholder interviews: about an hour
4. This information sheet will be provided to you.
5. If you join, you will be asked about your thoughts on the issue of sexual harassment. This issue may make you feel uncomfortable. You can stop participating or take breaks whenever you wish to do so.
6. This study is not intended to benefit you directly, but we hope this research will benefit people in the future.
7. Your privacy is very important to us. We will not collect your name for your participation unless it is needed for the activity. If it is needed, we will protect this information in a secure location and it will be accessible to key study staff and no data will ever be linked to your name.

**Thank you for your interest in our study. If you have any questions about this study or your part in it or you have questions, concerns or complaints about the research please contact:**

Dr. Aida Essaid, Director, Information and Research Center – King Hussein Foundation (IRCKHF); 06 0560 6010

Dr. Abeer Dababneh, Director of the University of Jordan’s Center for Women’s Studies (CFWS); 06 535 5000
