# Supplementary material for: Diagnosing Norms Surrounding Sexual Harassment at a Jordanian University
Source: Front Sociol. 2021 Jul 26;6:667220. doi: 10.3389/fsoc.2021.667220 (PMC8350132; doi:10.3389/fsoc.2021.667220)
Supplement: Supplementary file 2 [file Table2.docx]

| Name | Description | Excludes/Notes |
| --- | --- | --- |
| Forms of Sexual Harassment - Fitzgerald 1995 |  |  |
| Gender Harassment | Gender harassment refers to a broad range of verbal and nonverbal behaviors not aimed at sexual cooperation but that convey insulting, hostile, and degrading attitudes about women. Some examples include sexual epithets, slurs, taunts, and gestures; the display or distribution of obscene or pornographic materials; gender-based hazing; and threatening, intimidating, or hostile acts. 1) told suggestive stories 3) made crude sexual remarks 4) made offensive remarks 8) displayed offensive materials 9) sexist comments |  |
| Sexual Coercion | Sexual coercion constitutes the canonical example of sexual harassment, that is, the extortion of sexual cooperation in return for job-related considerations. Sexual coercion is the equivalent to the legal concept of quid pro quo 11) . . . subtly bribed you 12) . . . subtly threatened you 17) . . . made it necessary to cooperate to be well treated 18) . . . made you afraid of poor treatment if you didn't cooperate 19) . . . experienced consequences for refusing |  |
| Unwanted Sexual  Attention | Unwanted sexual attention includes a wide range of verbal and nonverbal behavior that is offensive, unwanted, and unreciprocated. 2) . . . attempted to discuss sex 5) . . . unwanted sexual attention 6) . . . staring, leering at you 7) . . . attempts to establish a sexual relationship 10) . . . repeated requests for drinks, dinner, despite rejection 13) . . . touching in a way that made you feel uncomfortable 14) . . . attempts to stroke or fondle | Code when “commenting” is mentioned |
| Other | Harassment items or tactics that could fall into multiple categories as described by Fitzgerald or they represent a different construct or form of sexual harassment. NOT for mentions of SH too vague to categorize (behaviour must be described in some detail). |  |
| Technology | Mention of the use of technology as a mode of SH. |  |
| Prevalence of Sexual Harassment on Campus | Perceptions of the amount of sexual harassment that is perpetrated on campus against males or females (empirical expectations). | Can be none |
| Location | The physical locations where sexual harassment takes place. | Must be university related in some way |
| On campus |  |  |
| Off campus |  | A university actor should still be involved to use this code |
| Time | Any reference to times of day that SH is most likely to occur |  |
| Laws | Discussion of whether laws or rules exist to prevent or punish sexual harassment on campus or in Jordan more widely |  |
| Explanations for SH | Factors which either explain why perpetrators harass or why survivors are harassed. Can also include factors which make people less likely to harass/be harassed. |  |
| Attitudes and Beliefs | Attitudes and beliefs that make people more likely to harass |  |
| Sex | Sex of perpetrator or victim is indicated as a risk factor | Mostly for “who are the victims?” type questions |
| Sexual Arousal | uncontrollable sexual arousal or desire is presented as an explanation for why some men perpetrate SH |  |
| Masculinity | Discussions of the link between masculinity or strict male gender norms and sexual harassment |  |
| Gender inequality | How lack of equality between the genders or power differential affects the perpetration of SH |  |
| Upbringing | Aspects of a person's upbringing that promote or prevent the perpetration of sexual harassment. Focused on the family or interpersonal relationships and not the wider social environment. | If mention is of family sanctioning/ending abuse, code as sanction not upbringing |
| Religion / Morals | Discussion of religion or morals as a risk or protective factor for SH |  |
| Conservative  Environment/Culture Shock | Discussion of how the attributes of conservativism (e.g., separation of the sexes, women confined to the traditional sphere) affect sexual harassment. Also includes the reaction that one feels coming into a new environment that has different social norms and behaviors around sex and sexual behavior and how this relates to SH |  |
| Dress | The ways in which a person's dress or apparel plays into how SH is perpetrated against her or how SH perpetrate against her/him is perceived. |  |
| Attractiveness of Survivor | the physical attractiveness of a woman (apart from dress) presented as an explanation for perpetration of SH |  |
| Impact of Sexual Harassment | Description of the physical, emotional, academic or other impact on survivors of sexual harassment. |  |
| Wider Impact | Impact beyond that on survivor to university or broader society |  |
| Survivor Response to Sexual Harassment | All aspects of victims responding to sexual harassment including how victims respond to SH; why they respond the way they do; and from whom they can or should seek help |  |
| Survivors immediate  Responses | How the survivor responds to the harasser immediately upon being harassed. Could include active responses and nonresponses (example does nothing). |  |
| Survivors Formal Help  Seeking | Descriptions of the survivors formal help seeking behaviors or the sources from whom she/he could seek help for sexual harassment (eg Dean’s office, campus police). | Does not include friends or family members which would be coded as informal coding |
| Survivors Informal Help  Seeking | Descriptions of the survivors Informal help seeking behaviours INCLUDING disclosure, or the sources from whom she/he could seek help or advice for sexual harassment. Generally includes individuals such as friends. | Excludes sources that are more formal in nature such as the police which would be coded as formal help seeking |
| Repercussions (and barriers) to reporting | Discussion of the potential barriers and responses to SH disclosure. Could include responses by family members, university staff, friends etc. |  |
| Rumours/reputation | Repercussion (either realized or hypothesized) includes mention of rumours or reputation. |  |
| Victim-Blaming | Reasons given for why female victims of SH are responsible for the SH they experience. |  |
| “Decent” | Mention of girls being decent or differentiating girls who are “decent” from those who are not. | Also includes synonyms for decent e.g. respectable, good girl |
| Bystander | The behaviors and responses of the person or people who are physically present when sexual harassment takes place. These people are not the perpetrator or the victim |  |
| Connected to Survivor | The bystander is mentioned as being with the survivor. |  |
| Connected to  Perpetrator | The bystander is mentioned as being with the perpetrator |  |
| Other | The bystander is mentioned without attribution to a particular party. |  |
| Norms |  |  |
| Empirical Expectations | Descriptive norms (what participants believe others do) |  |
| Normative Expectations | What participants (perceive others to) believe is right or acceptable |  |
| Exceptions | Examples of norms violations with or without sanctions |  |
| Reference groups | Groups that participants identify as key influencers of norms-related decision making |  |
| Peers |  |  |
| Family |  |  |
| Tribe |  |  |
| University personnel |  |  |
| Sanctions |  |  |
| Positive | approval, support, endorsement, encouragement, validation |  |
| Negative | penalty, reprisal, admonishment, discouragement |  |
| Lack of sanctions | Explicit mention that there are no sanctions. |  |
| Response of harasser to  sanction | Actual or hypothesized response of the harasser to the sanction. | Basically sensitivity to sanctions |
| Tribalism | The aspects of tribes or tribalism that affect sexual harassment. |  |
| Wasta | Role of having beneficial connections (example escaping punishment for sexual harassment because of these connections) |  |
| Flirting Vs SH | Discussion of flirting/appropriate sexual/romantic advances May be discussed alone or juxtaposed with SH. Includes aspects of behaviors as well as the initiator of the act. |  |
| Perceived consent | Mention that the survivor consented to the treatment explicitly or implicitly. |  |
| Institutional factors | Aspects of the physical environment, or university policies and practices, that influence the occurrence or reporting of SH. Should be UJ-specific. | e.g. bus system is overloaded, professors lock classrooms for late students |
| Gender/ Identity of institutional actor | A discussion of the institutional actor’s (employee/staff member/faculty) gender as an attribute that impacted sanctioning or reporting process |  |
| Data Collection | A discussion about how SH instances or reports are synthesized and communicated to higher level university actors, governments, or NGOs |  |
| Security | Security staff, discussion about the role of security in sanctioning SH |  |
| Specialized committees | Committees that investigate and adjudicate allocations of SH. |  |
| Policies and Procedures | Discusses the university’s written policies or codes, as well as normal procedures for reporting, adjudication, and punishment of complaints  Includes hypothetical discussions of how an instance of SH “would be” handled under university policy |  |
| Survivor support resources | Mental health counselling, support groups, etc. |  |
| Institutional Reputation | Discussion about how SH allegations impact the university’s reputation |  |
| Preventative actions | Education, awareness-focused events or communications about SH |  |
| Motivation for Reporting | Explanation for why an alleged survivor or hypothetical “reporter” is reporting SH | Includes both positive and negative motivators |
| False Accusation | Mention that an informal accusation or formal report of SH is false |  |
